# Lactose-Modified Hyaluronic Acid Molecule Attenuates In Vitro Chondrocyte Inflammation

**DOI:** 10.3390/cells14241977

**Published:** 2025-12-12

**Authors:** Alice Cristina Donato, Elisa Belluzzi, Valentina Masola, Pietro Ruggieri, Paola Brun

**Affiliations:** 1 Histology Unit, Department of Molecular Medicine (DMM), University of Padova, 35121 Padova, Italy; alicecristina.donato@studenti.unipd.it; 2 Department of Experimental Oncology, IEO, European Institute of Oncology IRCCS, Via Adamello 16, 20139 Milan, Italy; 3 Musculoskeletal Pathology and Oncology Laboratory, Department of Surgery, Oncology and Gastroenterology (DiSCOG), University of Padova, Via Giustiniani 3, 35128 Padova, Italy; elisa.belluzzi@unipd.it; 4 Orthopedics and Orthopedic Oncology, Department of Surgery, Oncology and Gastroenterology (DiSCOG), University Hospital of Padova, Via Giustiniani 3, 35128 Padova, Italy; 5 Department of Biomedical Sciences (DBS), University of Padova, 35121 Padova, Italy

**Keywords:** Osteoarthritis, hyaluronic acid, Hylach, inflammation, Galectin-3

## Abstract

Background: Osteoarthritis (OA) is a chronic degenerative whole joint disease characterized by cartilage breakdown and inflammation. Galectin-3 (Gal-3), a β-galactoside-binding lectin secreted into the extracellular space, binds to glycosylated components of the extracellular matrix (ECM), modulating cell–matrix interactions and inflammation. This study aims to evaluate the anti-inflammatory effects of Hylach^®^, a hyaluronic acid (HA) derivative conjugated with lactose-based residues that bind Gal-3, on in vitro inflamed primary human chondrocytes. Methods: Chondrocyte viability, after both Hylach^®^ and HA treatments at different concentrations was assessed using the MTT assay. Two-dimensional and 3D cell cultures exposed to the conditioned medium (CM) of activated U937 monocytes and subsequently treated with Hylach or HA, were analyzed for the expression of IL-1β, IL-6, TNF-α, and Gal-3 at different time points (4, 10, and 24 h). Results: HA and Hylach^®^ did not affect cell viability at any of the tested concentrations. Both molecules reduced the overexpression of Gal-3 and pro-inflammatory molecules in 2D inflamed cell cultures, at both gene and protein levels. Notably, IL-1β, IL-6 and Gal-3 showed a more pronounced inhibitory effect at 4 h, with Hylach demonstrating a stronger reduction compared to native HA. Moreover, in inflamed 3D chondrocyte cultures, Hylach^®^ but not HA, significantly reduced IL-1β, TNF-α and Gal-3 gene expression. Conclusions: Hylach^®^ exerts an early and more potent anti-inflammatory effect in inflamed 2D and 3D chondrocyte cultures when compared to HA. These findings suggest that targeting Gal-3 through selective HA derivatives may represent a promising strategy for modulating both inflammation and matrix remodelling in OA.

## 1. Introduction

Osteoarthritis (OA) is a chronic degenerative whole joint disease characterized by cartilage breakdown, meniscal degeneration, subchondral bone remodelling, inflammation and fibrosis of synovial membrane and infrapatellar fat pad [1,2,3,4]. OA represents a global cause of disability, affecting 7.96% of the global population in 2020 [5]. There is currently no cure for OA, and the disease remains a major cause of pain and disability worldwide. Management of OA focuses on symptom relief, functional improvement, and slowing disease progression. Current treatments include non-pharmacological interventions (i.e., patient education, weight loss, physical activity, and physical therapy) and pharmacological therapies (i.e., topical and oral NSAIDs, COX-2 inhibitors and intra-articular corticosteroids) [6,7]. Orthobiological therapies, such as platelet-rich plasma and mesenchymal stem cells, are emerging treatment options for OA [8]. However, robust evidence on their long-term efficacy and safety is still limited [9].

Conventional treatment with pain relievers and NSAIDs or corticosteroids can be effective, but it is often associated with significant side effects and toxicities. For this reason, viscosupplementation with hyaluronic acid (HA) has represented a notable advance in OA therapy, offering a local treatment option with a more favourable safety profile [10,11].

Hyaluronic acid (HA) is a naturally occurring glycosaminoglycan, consisting of repeating N-acetyl-D-glucosamine and D-glucuronic acid. It is found in the synovial fluid and the extracellular matrix (ECM) of articular cartilage, where it plays a key role in joint lubrication, shock absorption, and maintenance of the extracellular matrix [12,13,14,15]. In patients with OA, the concentration and molecular weight of endogenous HA is often reduced, contributing to joint stiffness, inflammation, and pain [16]. A wide array of HA formulations are currently used in clinical practice. These products differ in molecular weight (low, high, or ultra-high), degree of cross-linking, origin (avian-derived or bio-fermentation-based), dosage regimen (single, three, or five injections), and physicochemical properties. Moreover, some formulations contain non-crosslinked HA, while others are chemically crosslinked to enhance intra-articular residence time and therapeutic effect [17]. This heterogeneity can influence both efficacy and safety profiles, and there is currently no consensus on the most effective formulation.

**Figure 1:**
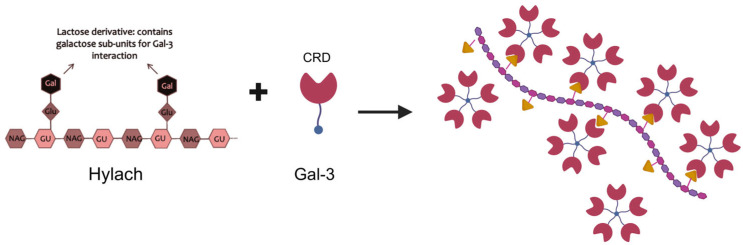
Schematic representation of the Hylach molecule and of its interaction with the CDR domain of Gal-3. In silico analyses provide a mechanistic rationale for the enhanced affinity of Hylach^®^ for Gal-3 showing that Hylach species with low to moderate lactosylation levels (approximately 30%) form highly stable and compact complexes with Gal-3. Energy contribution analyses further support these observations, demonstrating lower potential energy and more favourable binding energetics for these molecules, driven by increased van der Waals and hydrogen-bond contributions, particularly involving key carbohydrate recognition domain (CRD) residues [23].

A recent systematic review and meta-analysis including 169 clinical trials reported that visco-supplementation led to a small reduction in knee OA pain compared with placebo [18]. This variability, along with modest clinical benefit observed in some trials and meta-analysis, highlights the need to develop new and more effective HA-formulations to improve clinical outcomes for patients with OA. Recently, novel compounds have been proposed to enhance the therapeutic potential of HA, particularly by exerting additional anti-inflammatory and antioxidant effects. One such example is the combination of HA and chitosan modified with the addition of lactose, a ligand of the β-d-galactoside binding protein galectin, specifically recognized by its carbohydrate recognition domain (CRD) [19]. Both in vitro and in vivo studies showed the efficacy of this compound on cartilage regeneration and synovial inflammation [20,21]. Moreover, when the HA–Chitlac^®^ mixture was added to human chondrocytes treated with triamcinolone acetonide-hydroxypropyl-β-cyclodextrin, it markedly reduced the cytotoxic effects of the corticosteroid, while preserving its anti-inflammatory properties [22]. Recently, a lactose-modified HA (Hylach^®^) able to form highly stable and compact complexes with the pro-inflammatory molecule Galectin-3 (Gal-3, Figure 1), was shown to decrease macrophage-induced inflammation in human bronchial fibroblasts [23]. The same compound also demonstrated anti-fibrotic properties in lung fibroblasts [24]. Notably, Hylach^®^ has been reported to bind Gal-3 [19], a molecule also involved in cartilage ECM remodelling [25]. Since fibrosis is also a hallmark of OA [26], we hypothesized that Hylach^®^ may exert anti-inflammatory effects in OA chondrocytes as well. Therefore, the aim of this study is to evaluate the effects of Hylach^®^ on human primary chondrocytes exposed to conditioned medium (CM) from macrophages in two dimensional (2D) and three dimensional (3D) cultures, to simulate the inflammatory microenvironment typical of OA.

## 2. Materials and Methods

### 2.1. Compounds

Hylach^®^, a low molecular weight HA (ranging from 80 to 150 kDa) with a lactosylation degree of about 30%, lactose-derived residues, and HA was provided from GlycoCore Pharma (Como, Italy) [27]. The compounds were dissolved in saline phosphate buffer before use. Solutions were kept on ice during handling and stored at 4 °C when not in use, and all preparations were used within 24 h of dissolution to ensure stability.

### 2.2. Activated U937 Monocyte Conditioned Medium

The human monocyte U937 cell line was purchased from Thermo Scientific (Wilmington, DE, USA), cultured in RPMI (Euroclone, Pero, Italy) with 10% fetal bovine serum (FBS; Gibco, ThermoFisher, Waltham, MA, USA) and 1% antibiotics. To induce macrophage differentiation, U937 cells were treated with 50 ng/mL phorbol 12-myristate 13-acetate (PMA; Sigma-Aldrich, St. Louis, MO, USA) for 48 h. After differentiation, cells were exposed to 1 µg/mL lipopolysaccharide (LPS; Sigma-Aldrich, St. Louis, MO, USA) for 1 h. Macrophage differentiation was confirmed by observing cell morphology under inverted-phase contrast microscope and by assessing mRNA expression of CD68 and pro-inflammatory cytokines, as previously published [21,28] and presented in Supplementary Figure S1.

Subsequently, cells were rinsed and cultured for 24 h in complete RPMI medium to generate inflammatory conditioned medium (CM), which was then collected, centrifuged, filtered, and used to treat primary human chondrocyte cells and spheroids, as previously reported [21,28]. Cells were cultured at 37 °C with 5% CO_2_.

### 2.3. Human Primary Chondrocytes

Human articular chondrocytes were isolated from the femoral condyles and tibial plateau from knee OA patients (two males and one female; mean age 73 years; Kellgren-Lawrence grade 3) undergoing total knee replacement according to a standard protocol [29]. The study was approved by the local Ethical Committee (AOP1617), and all patients signed the informed consent. Tissue cartilage was finely minced and subjected to enzymatic digestion with 0.25% trypsin (Invitrogen, Carlsbad, CA, USA) for 15 min, followed by overnight incubation at 37 °C with type I collagenase (100 U/mL, Worthington Biochemical, Lakewood, NJ, USA). The resulting cell suspension was resuspended in Dulbecco’s Modified Eagle’s Medium (DMEM) supplemented with 10% FBS, 50 μg/mL L-ascorbic acid (Sigma, St. Louis, MO, USA), 1% penicillin/streptomycin (P/S), and 1.2% glutamine (all from Gibco, ThermoFisher, Waltham, MA, USA). The isolated cells were then expanded in culture at 37 °C with 5% CO_2_ with daily medium replacement, and were used for experiments only up to passage 3. Cell viability was assessed by trypan blue exclusion staining.

### 2.4. Formation and Cultivation of Human Chondrocyte Spheroids

To generate 3D chondrocyte spheroids, 10,000 cells were seeded in 3% Matrigel (Corning Inc., Corning, NY, USA) into U-bottom Ultra Low Adherence plates (Sarstedt AG & Co. KG, Nürnbrecht, Germany). The plates were then centrifuged at 600× *g* and incubated for 72 h at 37 °C, 5% CO_2_ to allow the formation of a single spheroid per well. The spheroids were then cultured in complete DMEM medium supplemented with 50 μg/mL L-ascorbic acid. Their morphology and size were monitored using a Leica phase contrast microscope (Leica Microsystems, Wetzlar, Germany), with bright-field images analyzed through ImageJ (version 1.54g; Fiji Is Just ImageJ, NIH, Bethesda, MD, USA).

### 2.5. Human Primary Chondrocytes and Spheroids Viability Assay

To investigate the impact of Hylach on chondrocyte cultures viability, cells derived from three independent donors were cultured in 2D and in 3D under standard conditions. Chondrocytes were plated in 2D at a density of 7000 cells/cm^2^ in 96-well plates and exposed to increasing concentrations of HA and Hylach (0.25; 0.5; 0.75; 1 and 1.25 mg/mL), for different durations. Cell viability was measured after 1, 3, and 6 days using the MTT assay (Sigma-Aldrich, St. Louis, MO, USA), based on a modified version of the protocol originally described by Denizot [30]. The assessment of cell spheroids viability was performed using propidium iodide (PI, Sigma-Aldrich, St. Louis, MO, USA) assay. Briefly, after removing the culture medium, the spheroids were washed with PBS, fixed in 4% paraformaldehyde for 10 min and PI was added for 15 to 30 min followed by DAPI (4′,6-diamidino-2-phenylindole) for 10 min to stain (in blue) all cell nuclei. Spheroids were then mounted on glass slides and analyzed using a Leica Stellaris 8 confocal microscope to assess their viability.

### 2.6. Analysis of Anti-Inflammatory Effects of Hylach on Primary Human 2D and 3D Chondrocyte Cultures

Human primary chondrocytes were cultured under both 2D and 3D conditions. For 2D cultures, 100,000 cells were seeded per well in 6-well plates. For 3D cultures, 10,000 cells per well were seeded in 96-well plates. After 3 days of culture, in order to mimic the inflammatory environment typical of OA, chondrocytes were exposed to U937-conditioned medium (CM): 2D cultures were treated for 24 h, while 3D cultures were treated for 48 h, as previously described in our published study [21].

Then, the cells in 2D or in 3D were washed and incubated with fresh culture medium containing HA 0.5 mg/mL or Hylach 0.5 mg/mL (based on viability experiments). Gene and protein expression of several inflammatory molecules were assessed by quantitative PCR and ELISA assays, respectively.

Total RNA was isolated from cells at 4-, 10-, and 24 h post-treatment in 2D and 3D culture using TRIzol reagent (Life Technologies, Carlsbad, CA, USA) according to the manufacturer’s protocol. Purity and concentration were assessed by measuring absorbance ratios at 260/280 nm with a Nanodrop 2000c spectrophotometer (Thermo Scientific, Waltham, MA, USA). To eliminate genomic DNA, DNase I (ThermoFisher, Waltham, MA, USA) treatment was performed. For complementary DNA (cDNA) synthesis, 500 ng of total RNA was reverse transcribed using oligo-dT primers and Superscript II reverse transcriptase (Life Technologies), following the manufacturer’s instructions. Quantitative PCR (qPCR) was performed on a Rotor-Gene RG-3000A instrument (QIAGEN, Hilden, Germany) using Xpert fast SYBR Green chemistry (GRISP, Porto, Portugal). The expression levels of IL-1beta, TNF-alpha, IL-6 and Gal-3 were evaluated. Expression of *Peptidylprolyl Isomerase A* was used as the housekeeping gene. mRNA expression was calculated. Gene expression was assessed using the 2^ΔCt^ method, in which ΔCt = Ct peptidylprolyl isomerase A (PPIA) − Ct target gene. The primer sequences used are reported in Table 1.

Supernatants from human primary chondrocytes inflamed by the CM from U937 cells and treated with HA or Hylach (both 0.5 mg/mL) were collected at different time points (4 h, 10 h and 24 h). TNF-α and IL-1β levels were measured using ELISA kits from R&D Systems (Minneapolis, MN, USA). IL-6 was quantified using a human ELISA kit from Boster Bio (Pleasanton, TX, USA), and Galectin-3 (Gal-3) was measured using an ELISA kit from Sino Biological (Wayne, PA, USA). Gene expression and protein measurements were performed on the same samples. All assays were performed according to the manufacturers’ protocols.

### 2.7. Statistical Analysis

Each experiment was performed in triplicate. Data are reported as the mean and the standard error of the mean (SE). Statistical analyses and graphs were performed using GraphPad Prism 10 (GraphPad Inc., San Diego, CA, USA). Data were analyzed by one-way ANOVA followed by Tukey’s multiple comparison test or by unpaired Student’s *t*-test, as appropriate. A *p* < 0.05 was considered statistically significant.

## 3. Results

### 3.1. Chondrocyte’s Viability in 2D Is Not Affected by HA or Hylach Treatment

**Figure 2:**
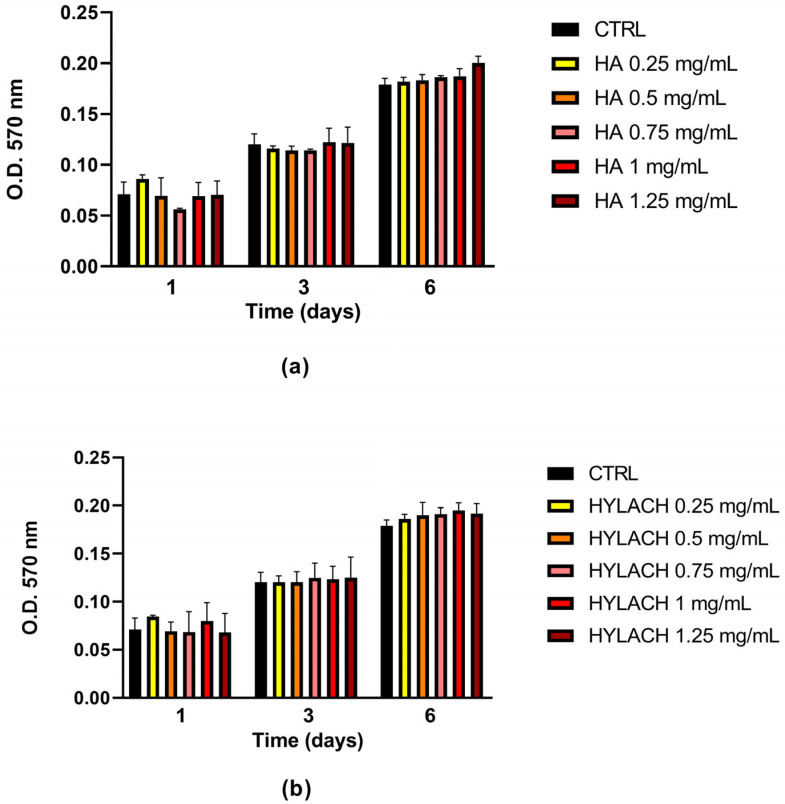
Effects of HA and Hylach on chondrocyte viability. Chondrocytes were seeded at a density of 7000 cells/cm^2^ in 96-well culture plates and treated with different concentrations of (**a**) hyaluronic acid (HA) or (**b**) Hylach. Cell viability was assessed using the MTT assay after 1, 3, and 6 days of treatment. Data are reported as mean ± SE of three independent experiments. Statistical significance was determined using one way ANOVA.

Human primary chondrocytes were exposed to increasing concentrations of HA and Hylach to evaluate potential effects on cell viability. Assessments were conducted after 1, 3, and 6 days of treatment. According to the results of the MTT assay, no significant differences in cell viability were observed in treated samples compared to untreated controls (Figure 2) and among donors.

Based on these data and previously published studies [23,24], we decided to use HA or Hylach 0.5 mg/mL for subsequent experiments.

### 3.2. Hylach Attenuates Inflammation in Human Primary Chondrocytes Inflamed by the CM of Macrophage

**Figure 3:**
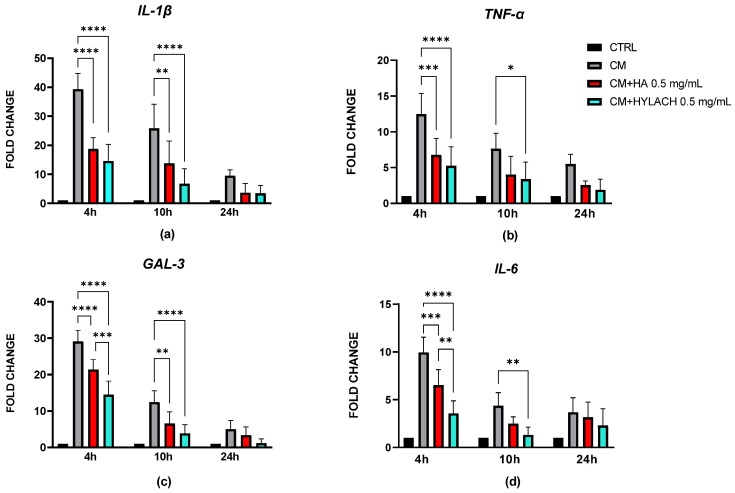
Gene expression analysis by real-time PCR in chondrocytes treated with HA or Hylach. mRNA expression levels of IL-1β (**a**), TNF-α (**b**), Galectin-3 (**c**), and IL-6 (**d**) were quantified by real-time PCR in chondrocytes pretreated with conditioned medium (CM) from activated macrophages and subsequently treated with hyaluronic acid (HA) or Hylach. Results are shown as mean ± SD from three independent experiments. Statistical significance was determined using one way ANOVA. Asterisks indicate statistically significant differences: * *p* < 0.05; ** *p* < 0.01; *** *p* < 0.001 and **** *p* < 0.0001.

**Figure 4:**
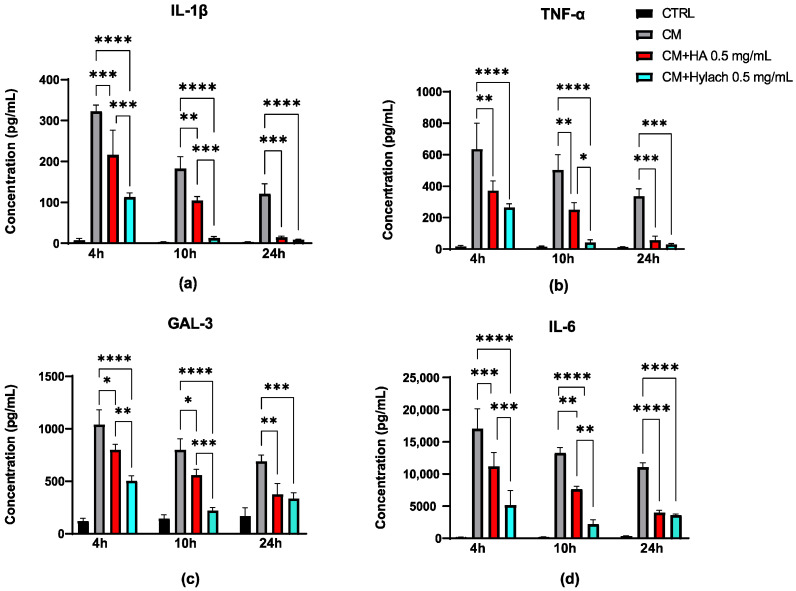
Evaluation of protein expression of inflammatory cytokines in chondrocytes treated with HA or Hylach. Protein expression levels of IL-1β (**a**), TNF-α (**b**), Gal-3 (**c**), and IL-6 (**d**) were quantified by ELISA assays in chondrocytes pretreated with conditioned medium (CM) from activated macrophages and subsequently treated with hyaluronic acid (HA) or Hylach. Statistical significance was determined using one way ANOVA. Data are reported as mean ± SE of three independent experiments. Asterisks indicate statistically significant differences: * *p* < 0.05; ** *p* < 0.01; *** *p* < 0.001 and **** *p* < 0.0001.

Human primary chondrocytes cultured in 2D were pretreated with CM from U937 cells to mimic the inflammatory environment of OA. The treatment induced a significant increase in the expression of *IL-1β*, *TNF-α*, *IL-6*, and *Gal-3*, reflecting an inflammatory and catabolic phenotype typical of OA (Figure 3 and Figure 4). Following CM stimulation, the inflamed cells were treated with HA or Hylach to assess their effects on inflammatory cytokines and *Gal-3* at different time points. At 4 h post-treatment, both HA and Hylach significantly reduced the mRNA expression of *IL-1β*, *TNF-α*, *Gal-3*, and *IL-6* compared to CM-treated controls, indicating an attenuation of the inflammatory response (Figure 3). At 10 h, a significant decrease in *IL-1β* and *Gal-3* mRNA expression was still observed in both HA- and Hylach-treated cells, whereas a reduction in TNF-α and IL-6 expression was evident only in the Hylach-treated group. After 24 h, although a downward trend in the expression of all evaluated genes persisted, no statistically significant differences were detected.

Protein expression analysis confirmed the mRNA results at 4 h post-treatment (Figure 4). At both 10 and 24 h, the decrease in protein levels remained statistically significant for both HA and Hylach treatments. Moreover, Hylach showed a greater ability to reduce IL-1β, Gal-3, and IL-6 protein levels at both 4 and 10 h compared to HA. A marked reduction in the expression of these proteins was observed at these time points, with Hylach being significantly more effective than HA (*p* < 0.01). As for *TNF-α*, a significant difference between the treatments was observed only at the 10 h time point.

### 3.3. The Anti-Inflammatory Effect of Hylach on Inflamed Chondrocytes Was Confirmed in a 3D Spheroid Model

**Figure 5:**
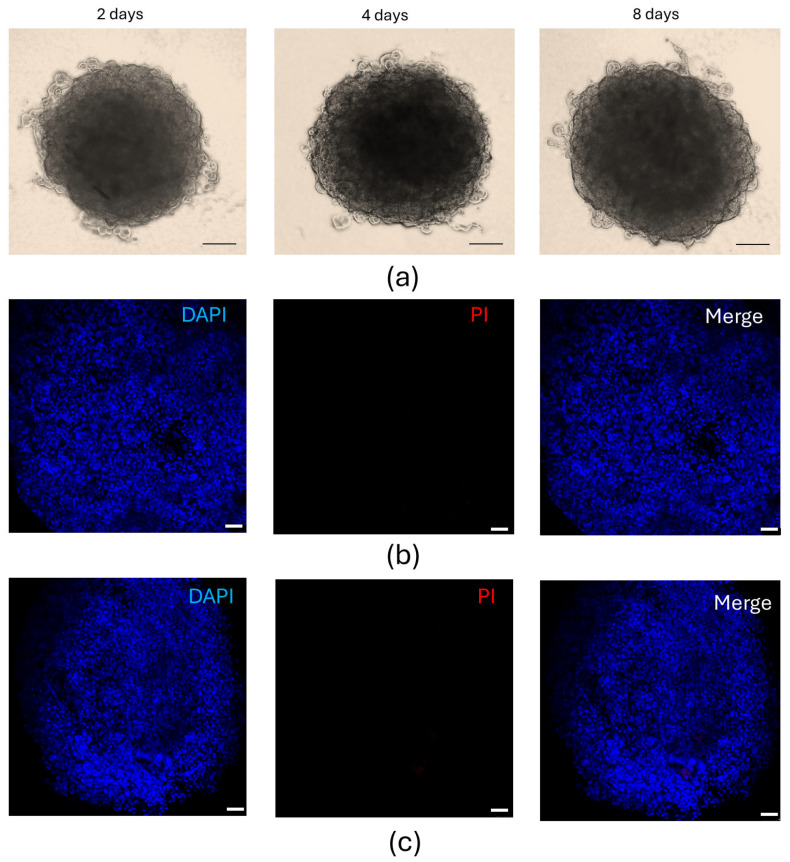
Whole-mount human chondrocyte spheroid viability. (**a**) Bright-field microscopy images showing chondrocyte spheroids over an 8-day culture period. The scale bar represents 200 µm. (**b**,**c**) Nuclear (DAPI) and propidium iodide (PI) staining of the spheroids untreated (**b**) or treated (**c**) with the CM of U937 activated monocytes for 48 h. The absence of PI-positive nuclei indicates that all cells within the spheroids are viable. The scale bar represents 50 µm. Data are reported as mean ± SE of three independent experiments.

After performing experiments on 2D cell cultures, we next moved to a 3D model using spheroids. Figure 5 shows spheroids observed under bright-field microscopy from 2 to 8 days of culture and the assessment of cell viability both before and after treatment with the CM of activated U937 cells for 48 h to mimic OA microenvironment.

**Figure 6:**
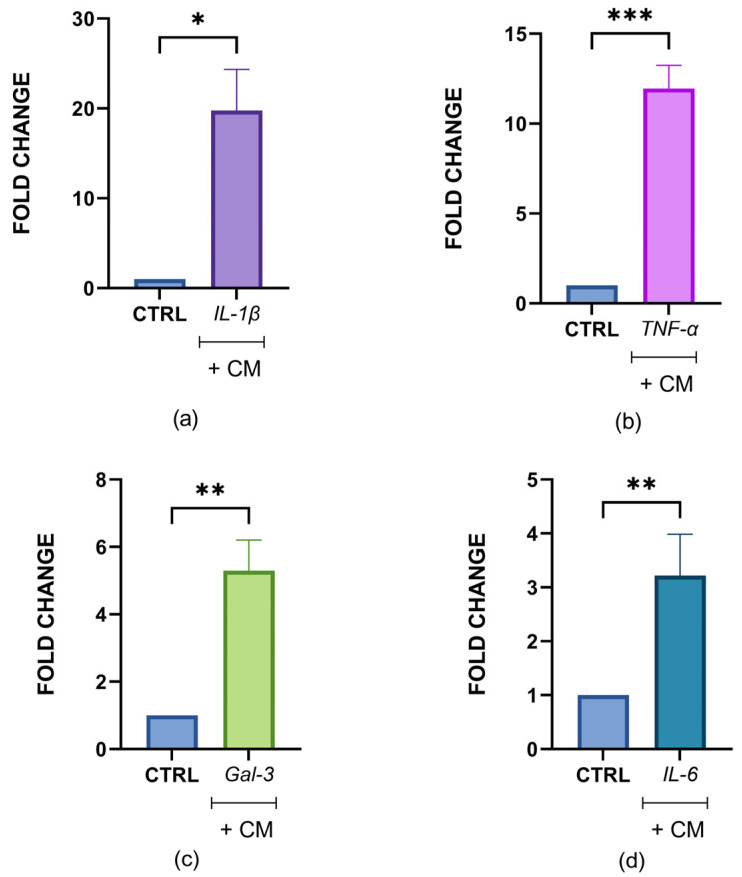
Gene expression analysis in real-time PCR in spheroids treated with activated U937 CM for 48 h. Gene expression of IL-1β (**a**), TNF-α (**b**), Gal-3 (**c**), and IL-6 (**d**) was quantified by real-time PCR in spheroids treated with conditioned medium (CM) of activated U937 cells. Statistical significance was determined using the unpaired *t*-test. Asterisks indicate statistically significant differences: * *p* < 0.05; ** *p* < 0.01 and *** *p* < 0.001.

Subsequently, the qPCR analyses performed after treatment of chondrocyte spheroids with CM demonstrated an increase in *IL-1β*, *TNF-α*, *Gal-3*, and *IL-6* gene expression (*p* < 0.05) in all the chondrocyte spheroids (Figure 6).

**Figure 7:**
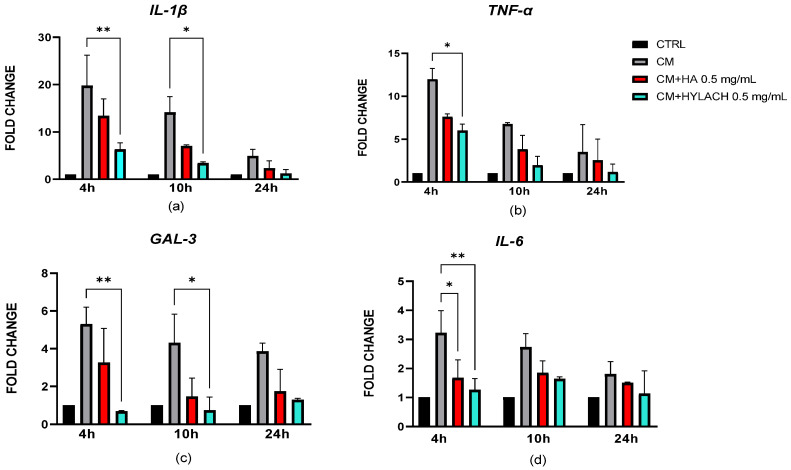
Gene expression analysis with real-time PCR in spheroids treated with HA or Hylach. mRNA expression levels of IL-1β (**a**), TNF-α (**b**), Gal-3 (**c**), and IL-6 (**d**) were quantified in real-time PCR in spheroids pretreated with conditioned medium (CM) from activated macrophages and subsequently treated with hyaluronic acid (HA) or Hylach. Results are shown as mean ± SD from three independent experiments. Statistical significance was determined using one way ANOVA. Asterisks indicate statistically significant differences: * *p* < 0.05 and ** *p* < 0.01.

The effect of native HA and Hylach with 30% of lactosylation on inflamed human chondrocytes, following a 48 h exposure to activated U937 CM, was evaluated at various time intervals using chondrocyte spheroids, after 3 days of culture. The expression of pro-inflammatory molecules *IL-1β*, *Gal-3*, *IL-6* and *TNF-α*, assessed at the gene level confirmed the results obtained with 2D cultures, as shown in Figure 7.

Hylach was able to downregulate the gene expression of *IL-1 β*, *TNF*-α, *IL-6*, and *Gal-3* as early as 4 h after treatment, whereas HA did not exert this effect. However, both HA and Hylach reduced IL-6 expression levels at 4 h, with a significantly stronger effect observed for Hylach (*p* < 0.01). The downregulation effect was not maintained at 10 and 24 h for *TNF*-α and *IL-6*, while *IL-1β* and *Galectin-3* expression remained reduced at 10 h following Hylach treatment.

## 4. Discussion

In this study, we demonstrated that Hylach a lactose-modified HA able to bind to Gal-3, exerts significant anti-inflammatory effects on primary human chondrocytes exposed to the macrophage-conditioned medium (CM), one of the main cell drivers of OA progression. In fact, it has been demonstrated that the two main cytokines released by macrophages, IL-1β and TNF-α, are able to induce the resumption of chondrocyte metabolism [31]. Notably, Hylach reduced the expression not only of Gal-3, but also that of key pro-inflammatory cytokines (IL-1β, TNF-α, IL-6) and Gal-3 more effectively and rapidly than HA, particularly at the early time point of 4 h. These findings were consistent both at the mRNA and protein level and were further confirmed in 3D chondrocyte spheroid cultures. The choice to investigate Hylach molecules with a 30% of lactose-derived residues at a concentration of 0.5 mg/mL in the context of OA was based on prior evidence showing its ability to bind Gal-3 and reduce inflammation in bronchial and pulmonary fibroblasts [23,24] downregulating the expression of pro-inflammatory cytokines and of galectins.

Gal-3, a β-galactoside-binding lectin that plays a central role in ECM remodelling and inflammation, is overexpressed in OA chondrocytes and acts in combination with other pro-inflammatory molecules [25,32]. Secreted Gal-3 induces MMP-3 and ADAMTS5 expression in chondrocytes exacerbating ECM degradation [32,33]. Therefore, modulating the expression of this molecule is of particular interest in counteracting OA. Moreover, given that the clinical efficacy of intra-articular HA in OA remains a matter of ongoing debate [34], with major guidelines questioning its routine use [35,36], there is a pressing need to develop novel formulations with enhanced biological activity. Strategies aimed at improving the anti-inflammatory and disease-modifying potential of HA are particularly relevant, especially in patients with inflammation-driven OA phenotypes. In the literature, several mixtures of HA with different molecules showed to be promising for OA [37,38]. Tarricone et al. showed that HA mixed with Chitlac (HA-CTL) was able to neutralize the up-regulation of *IL-1β*, *TNF-α*, *Gal-1* and *MMP* gene expressions in human primary chondrocytes treated with CM from macrophage [21]. The efficacy was confirmed in a rat knee OA model [20]. The mixture HA-CTL has also been shown to attenuate the cytotoxicity induced by triamcinolone acetonide (TA) [22], one of the most used corticosteroids, in combination with cyclodextrin (CD), a cyclic oligomer of glucose used as a drug carrier to enhance their solubility [39], in chondrocytes, while maintaining the anti-inflammatory effects of TA [22].

Our results support the hypothesis that targeting Gal-3 via lactose-based HA derivatives could be a promising strategy for modulating inflammatory responses in OA. Hylach was not cytotoxic at any of the tested concentrations, indicating a favourable safety profile similar to that of unmodified HA. Furthermore, the use of both 2D and 3D models in our study adds robustness to the findings and allows better simulation of the in vivo chondrocyte niche. In contrast, HA alone exhibited milder effects. This difference may be attributed to the specific structural modifications of Hylach, which likely enhance its interaction with Gal-3 and possibly other ECM or cell surface components involved in inflammation.

Our study also highlights the importance of time-course evaluation: the peak anti-inflammatory activity was observed at 4 h, suggesting that Hylach acts rapidly upon administration. This temporal profile mirrors our previously published observations for modified-HA systems (i.e., HA-Chitlac), where rapid modulation occurs soon after exposure, followed by a gradual decrease in effect at later time points. The rapid and pronounced reduction in inflammatory markers after Hylach treatment suggests a potential for early intervention in acute inflammatory phases of OA. While the anti-inflammatory effect declined over time, a trend toward reduced cytokine and Gal-3 expression persisted also at later time points. Sustained inhibition likely requires prolonged presence or repeated dosing, which should be investigated in future in vitro and in vivo studies.

Several limitations should be acknowledged. First, this is an in vitro study, and although primary human chondrocytes were used, the relevance of 2D models in the contest of disease pathophysiology has to be interpreted with caution. Nevertheless, we use a 3D culture system model to confirm our 2D results in a more physiologically relevant context, and the use of 3D models in OA studies is considered of relevance to reduce the gap between cell culture and in vivo conditions [40]. Second, we did not assess the long-term effects or regenerative potential of Hylach, such as ECM synthesis or matrix metalloproteinase activity. Third, while we focused on Gal-3 due to its known interaction with β-galactoside residues, other potential molecular pathways modulated by Hylach remain to be investigated. However, our previous in silico findings (Figure 1 [23]) may suggest that Hylach’s multivalent display of lactose residues enables simultaneous engagement of multiple sites on Gal-3, resulting in a more stable and higher-affinity complex compared with native HA. By occupying key binding sites in the CDR, Hylach^®^ may prevent Gal-3 from forming oligomeric clusters on the cell surface required to trigger downstream pro-inflammatory signalling through pathways including NF-κB and MAPKs.

Further studies are warranted to validate these effects in vivo and to clarify whether Hylach can modulate ECM homeostasis and support cartilage repair. In addition, mechanistic investigations will be essential to determine the specific contribution of Gal-3 binding to the observed responses.

In conclusion, our data suggest that Hylach may provide enhanced anti-inflammatory activity compared to standard HA and could represent a novel therapeutic tool for OA management.

## Figures and Tables

**Table 1:** Primer sequences.

Gene (Accession Number)	Name	Primer Sequences
*IL-1β* (NM_000576.3)	*Interleukin 1 beta*	Fw 5′-GAATCTCCGACCACCACTACAG-3′ Rv 5′-TGATCGTACAGGTGCATCGTG-3′
*LSGALS3* (NM_002306.4)	*Galectin 3*	Fw 5′-CTGCTGGGGCACTGATTGT-3′ Rv 5′-TGTTTGCATTGGGCTTCACC-3′
*PPIA* (NM_021130.5)	*Peptidylprolyl Isomerase A*	Fw 5′-GGGCTTTAGGCTGTAGGTCAA-3′ Rv 5′-AACCAAAGCTAGGGAGAGGC-3′
*TNF-α* (NM_000594.3)	*TNF-alpha*	Fw 5′-AAGCCTGTAGCCCATGTTGT-3′ Rv 5′-GGACCTGGGAGTAGATGAGGT-3′
*IL-6* (NM_000501.4)	*Interleukin 6*	Fw 5′-ATGAACTCCTTCTCCACAAGCG-3′ Rv 5′-CTCCTTTCTCAGGGCTGAG-3′

## Data Availability

The data presented in this study are available on request from the corresponding author.

## Supplementary Materials

The following supporting information can be downloaded at: https://www.mdpi.com/article/10.3390/cells14241977/s1, Figure S1: Activated U937 cells upregulate the expression of pro-inflammatory molecules.

## Abbreviations

The following abbreviations are used in this manuscript:

OA	Osteoarthritis
HA	Hyaluronic Acid
CM	Conditioned Medium
Gal-3	Galectin-3

## References

[1] Donell S. (2019). Subchondral Bone Remodelling in Osteoarthritis. EFORT Open Rev..

[2] Poole A.R. (2012). Osteoarthritis as a Whole Joint Disease. HSS J..

[3] Ozeki N., Koga H., Sekiya I. (2022). Degenerative Meniscus in Knee Osteoarthritis: From Pathology to Treatment. Life.

[4] Sanchez-Lopez E., Coras R., Torres A., Lane N.E., Guma M. (2022). Synovial Inflammation in Osteoarthritis Progression. Nat. Rev. Rheumatol..

[5] Steinmetz J.D., Culbreth G.T., Haile L.M., Rafferty Q., Lo J., Fukutaki K.G., Cruz J.A., Smith A.E., Vollset S.E., Brooks P.M. (2023). Global, Regional, and National Burden of Osteoarthritis, 1990–2020 and Projections to 2050: A Systematic Analysis for the Global Burden of Disease Study 2021. Lancet Rheumatol..

[6] Richard M.J., Driban J.B., McAlindon T.E. (2023). Pharmaceutical Treatment of Osteoarthritis. Osteoarthr. Cartil..

[7] Murphy S.L., Robinson-Lane S.G., Niemiec S.L.S. (2016). Knee and Hip Osteoarthritis Management: A Review of Current and Emerging Non-Pharmacological Approaches. Curr. Treat. Options Rheumatol..

[8] Shtroblia V., Petakh P., Kamyshna I., Halabitska I., Kamyshnyi O. (2025). Recent Advances in the Management of Knee Osteoarthritis: A Narrative Review. Front. Med..

[9] Budhiparama N.C., Putramega D., Lumban-Gaol I. (2024). Orthobiologics in Knee Osteoarthritis, Dream or Reality?. Arch. Orthop. Trauma Surg..

[10] Bellamy N., Campbell J., Robinson V., Gee T., Bourne R., Wells G. (2006). Viscosupplementation for the Treatment of Osteoarthritis of the Knee. Cochrane Database Syst. Rev..

[11] Abate M., Pulcini D., Di Iorio A., Schiavone C. (2010). Viscosupplementation with Intra-Articular Hyaluronic Acid for Treatment of Osteoarthritis in the Elderly. Curr. Pharm. Des..

[12] Sprott H., Fleck C. (2023). Hyaluronic Acid in Rheumatology. Pharmaceutics.

[13] Gupta R.C., Lall R., Srivastava A., Sinha A. (2019). Hyaluronic Acid: Molecular Mechanisms and Therapeutic Trajectory. Front. Vet. Sci..

[14] Fallacara A., Baldini E., Manfredini S., Vertuani S. (2018). Hyaluronic Acid in the Third Millennium. Polymers.

[15] Liao Y.-H., Jones S.A., Forbes B., Martin G.P., Brown M.B. (2005). Hyaluronan: Pharmaceutical Characterization and Drug Delivery. Drug Deliv..

[16] Super J.T., Makaram N.S., LaPrade R.F., Murray I.R. (2025). Intra-Articular Injections for the Management of Knee Osteoarthritis. Orthop. Trauma.

[17] Costa F.R., Costa Marques M.R., Costa V.C., Santos G.S., Martins R.A., Santos M.d.S., Santana M.H.A., Nallakumarasamy A., Jeyaraman M., Lana J.V.B. (2023). Intra-Articular Hyaluronic Acid in Osteoarthritis and Tendinopathies: Molecular and Clinical Approaches. Biomedicines.

[18] Pereira T.V., Jüni P., Saadat P., Xing D., Yao L., Bobos P., Agarwal A., Hincapié C.A., da Costa B.R. (2022). Viscosupplementation for Knee Osteoarthritis: Systematic Review and Meta-Analysis. BMJ.

[19] Argüeso P., Panjwani N. (2011). Focus on Molecules: Galectin-3. Exp. Eye Res..

[20] Salamanna F., Giavaresi G., Parrilli A., Martini L., Nicoli Aldini N., Abatangelo G., Frizziero A., Fini M. (2019). Effects of Intra-Articular Hyaluronic Acid Associated to Chitlac (Arty-Duo^®^) in a Rat Knee Osteoarthritis Model. J. Orthop. Res. Off. Publ. Orthop. Res. Soc..

[21] Tarricone E., Mattiuzzo E., Belluzzi E., Elia R., Benetti A., Venerando R., Vindigni V., Ruggieri P., Brun P. (2020). Anti-Inflammatory Performance of Lactose-Modified Chitosan and Hyaluronic Acid Mixtures in an In Vitro Macrophage-Mediated Inflammation Osteoarthritis Model. Cells.

[22] Tarricone E., Elia R., Mattiuzzo E., Faggian A., Pozzuoli A., Ruggieri P., Brun P. (2021). The Viability and Anti-Inflammatory Effects of Hyaluronic Acid-Chitlac-Tracimolone Acetonide-β-Cyclodextrin Complex on Human Chondrocytes. Cartilage.

[23] Donato A., Fontana F., Venerando R., Di Stefano A., Brun P. (2023). The Anti-Inflammatory Effect of Lactose-Modified Hyaluronic Acid Molecules on Primary Bronchial Fibroblasts of Smokers. Polymers.

[24] Donato A., Di Stefano A., Freato N., Bertocchi L., Brun P. (2023). Inhibition of Pro-Fibrotic Molecules Expression in Idiopathic Pulmonary Fibrosis-Derived Lung Fibroblasts by Lactose-Modified Hyaluronic Acid Compounds. Polymers.

[25] Toegel S., Bieder D., André S., Kayser K., Walzer S.M., Hobusch G., Windhager R., Gabius H.-J. (2014). Human Osteoarthritic Knee Cartilage: Fingerprinting of Adhesion/Growth-Regulatory Galectins in Vitro and in Situ Indicates Differential Upregulation in Severe Degeneration. Histochem. Cell Biol..

[26] Rim Y.A., Ju J.H. (2020). The Role of Fibrosis in Osteoarthritis Progression. Life.

[27] Nizzolo S., Esposito E., Ni M.-H., Bertocchi L., Bianchini G., Freato N., Zanzoni S., Guerrini M., Bertini S. (2024). A Novel Biomimetic Probe for Galectin-3 Recognition: Chemical Synthesis and Structural Characterization of a β-Galactose Branched Sodium Hyaluronate. Proteoglycan Res..

[28] Mattiuzzo E., Faggian A., Venerando R., Benetti A., Belluzzi E., Abatangelo G., Ruggieri P., Brun P. (2021). In Vitro Effects of Low Doses of β-Caryophyllene, Ascorbic Acid and d-Glucosamine on Human Chondrocyte Viability and Inflammation. Pharmaceuticals.

[29] Brun P., Abatangelo G., Radice M., Zacchi V., Guidolin D., Daga Gordini D., Cortivo R. (1999). Chondrocyte Aggregation and Reorganization into Three-Dimensional Scaffolds. J. Biomed. Mater. Res..

[30] Denizot F., Lang R. (1986). Rapid Colorimetric Assay for Cell Growth and Survival. Modifications to the Tetrazolium Dye Procedure Giving Improved Sensitivity and Reliability. J. Immunol. Methods.

[31] Glyn-Jones S., Palmer A.J.R., Agricola R., Price A.J., Vincent T.L., Weinans H., Carr A.J. (2015). Osteoarthritis. Lancet Lond. Engl..

[32] Weinmann D., Schlangen K., André S., Schmidt S., Walzer S.M., Kubista B., Windhager R., Toegel S., Gabius H.-J. (2016). Galectin-3 Induces a Pro-Degradative/Inflammatory Gene Signature in Human Chondrocytes, Teaming Up with Galectin-1 in Osteoarthritis Pathogenesis. Sci. Rep..

[33] Hu Y., Yéléhé-Okouma M., Ea H.-K., Jouzeau J.-Y., Reboul P. (2017). Galectin-3: A Key Player in Arthritis. Jt. Bone Spine.

[34] Glinkowski W.M., Tomaszewski W. (2025). Intra-Articular Hyaluronic Acid for Knee Osteoarthritis: A Systematic Umbrella Review. J. Clin. Med..

[35] Kolasinski S.L., Neogi T., Hochberg M.C., Oatis C., Guyatt G., Block J., Callahan L., Copenhaver C., Dodge C., Felson D. (2020). 2019 American College of Rheumatology/Arthritis Foundation Guideline for the Management of Osteoarthritis of the Hand, Hip, and Knee. Arthritis Care Res..

[36] Uson J., Rodriguez-García S.C., Castellanos-Moreira R., O’Neill T.W., Doherty M., Boesen M., Pandit H., Parera I.M., Vardanyan V., Terslev L. (2021). EULAR Recommendations for Intra-Articular Therapies. Ann. Rheum. Dis..

[37] Kim Y.S., Guilak F. (2022). Engineering Hyaluronic Acid for the Development of New Treatment Strategies for Osteoarthritis. Int. J. Mol. Sci..

[38] Walvekar P., Lulinski P., Kumar P., Aminabhavi T.M., Choonara Y.E. (2024). A Review of Hyaluronic Acid-Based Therapeutics for the Treatment and Management of Arthritis. Int. J. Biol. Macromol..

[39] Uekama K., Hirayama F., Arima H. (2006). Recent Aspect of Cyclodextrin-Based Drug Delivery System. J. Incl. Phenom. Macrocycl. Chem..

[40] Samvelyan H.J., Hughes D., Stevens C., Staines K.A. (2021). Models of Osteoarthritis: Relevance and New Insights. Calcif. Tissue Int..

