# Supplementary material for: Lactose-Modified Hyaluronic Acid Molecule Attenuates In Vitro Chondrocyte Inflammation
Source: Cells. 2025 Dec 12;14(24):1977. doi: 10.3390/cells14241977 (PMC12731145; doi:10.3390/cells14241977)
Supplement: Supplementary file 1 [file cells-14-01977-s001.zip › cells-3990877-supplementary.pdf]

Supplementary file

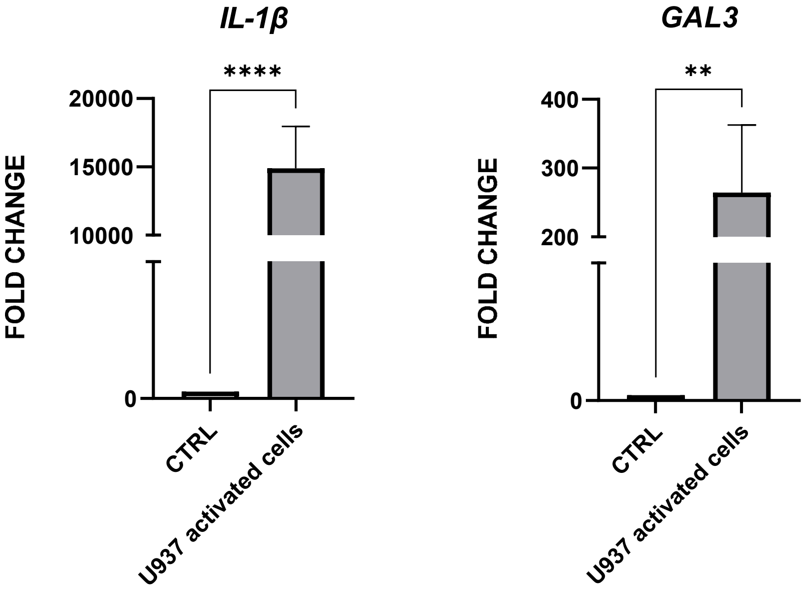

Figure S1. Activated U937 cells upregulate the expression of pro-inflammatory molecules. *U937 cells were stimulated with PMA and LPS for 24 hours and then cultured under optimal conditions. RNA transcript levels of IL-1β, and Gal-3 were analyzed by qPCR. Data are presented as mean ± SE from three independent experiments. Statistical significance was determined using an unpaired Student's* t*-test. ** P < 0.01, **** P < 0. 001 vs control (CTRL).*
